# Editorial: Sex-dependent modulation of neuroinflammation in the aging brain

**DOI:** 10.3389/fnagi.2025.1745448

**Published:** 2025-12-04

**Authors:** Yu-Min Kuo, Shun-Fen Tzeng, Celia Giulietta Fernandez

**Affiliations:** 1 Department of Cell Biology and Anatomy, College of Medicine, National Cheng Kung University, Tainan, Taiwan; 2 Department of Life Sciences, College of Biological Sciences and Technology, National Cheng Kung University, Tainan, Taiwan; 3 Recursion Pharmaceuticals, Salt Lake City, UT, United States

**Keywords:** sex differences, neuroinflammation, astrocyte-microglia interactions, estrogen signaling, gut-brain axis, aging and neurodegeneration

Neuroinflammation has emerged as a dynamic and multifactorial process that is central to both normal brain aging and the progression of neurodegenerative diseases. Recent discoveries reveal that the molecular and cellular mechanisms underlying neuroinflammatory responses are profoundly influenced by biological sex, shaping disease susceptibility, progression, and treatment outcomes. This Research Topic of review articles aims to summarize the current state of the field and guide future directions to understand how sex differentially impacts neuroinflammatory processes.

The Opinion article “*Regulatory mechanisms of neuroinflammation from a gender perspective: interactions among astrocytes, sex hormones, and the gut-brain axis*” (Shi et al.) explores how astrocytes integrate hormonal and gut-derived signals to coordinate sex-specific immune responses. Circulating estrogen and testosterone regulate astrocytic cytokine and chemokine release, thereby modulating microglial activity and local inflammatory tone. With the advent of single-cell sequencing, sex-dependent transcriptional networks within astrocytes are now being delineated, revealing unique regulatory nodes for each sex. The review further highlights how gut microbiota composition, modulated by sex hormones, affects metabolite production and brain immune signaling, adding another layer of complexity to sex-specific neuroimmune regulation. A picture emerges in which distinct, sex-specific cellular pathways are implicated in disease, suggesting that different treatment options may be appropriate depending on the sex of the patient.

In “*The estrogen–brain interface in neuroinflammation: a multidimensional mechanistic insight*” (Lu et al.), estrogen is described as a potent neuroprotective modulator acting through receptors on astrocytes, microglia, and neurons. By engaging key signaling cascades, including PI3K/Akt, NF-κB, and WNT/β-catenin, estrogen fine-tunes neuroimmune gene expression, suppressing pro-inflammatory pathways and promoting resolution. Beyond immunomodulation, estrogen enhances mitochondrial performance, safeguards neuronal DNA integrity, and stabilizes neural–glial interactions, thereby supporting homeostasis during aging. This systematic review further describes the bidirectional connection between estrogen and the gut microbiome. Distinct responses to various estrogenic compounds (e.g., 17α-estradiol) underscore the precision needed when leveraging these pathways therapeutically.

The mini-review “*Influence of biological sex on neuroinflammatory dynamics in the aging brain*” (Müller et al.) synthesizes hormonal, genetic, and epigenetic mechanisms underlying chronic glial activation, a defining feature of brain aging. Age-related neuroinflammation involves transcriptional and epigenetic reprogramming that shifts the balance toward a pro-inflammatory state. These changes are tightly linked to the hormonal transitions of menopause and andropause, which reshape glial function and immune surveillance in a sex-dependent manner.

Finally, “*Sex differences in the outcomes of modifiable lifestyle factors for cognitive aging: neuroinflammation and microglia as key underlying mechanisms*” (Coleborn et al.) focuses on the responsiveness of microglia to lifestyle interventions such as exercise, diet, and social activity. These behaviors elicit sex-specific changes in microglial activation, synaptic remodeling, and neurotrophic support, ultimately shaping neuroinflammatory trajectories during aging.

Together, these contributions highlight the necessity of integrating sex as a key biological variable in mechanistic and translational studies of neuroinflammation. Advances in genomics, epigenetics, and cell-based modeling are now enabling the identification of sex-specific therapeutic targets within glial networks and immune signaling pathways. The development of patient-derived brain organoids and co-cultures from both sexes, as well as sex-stratified clinical trials and AI/ML modeling methods, promises to accelerate the translation of these findings into precision therapeutics.

A systems-level framework, combining longitudinal profiling, network biology, and hormonal analysis, will be crucial to unravel how sex, glial cell dynamics, and environmental inputs interact to shape complex neuroinflammatory outcomes. Such mechanistic understanding will guide the design of targeted interventions and preventative strategies tailored to the distinct neurobiological needs of aging men and women.

